# Faecal markers of intestinal inflammation in slum infants following yogurt intervention: A pilot randomized controlled trial in Bangladesh

**DOI:** 10.3389/frmbi.2023.1029839

**Published:** 2023-03-23

**Authors:** Kaniz Jannat, Md. Abdul Kader, Sarker Masud Parvez, Russell Thomson, Mahbubur Rahman, Mamun Kabir, Kingsley Agho, Rashidul Haque, Dafna Merom

**Affiliations:** ^1^ School of Health Sciences/Centre for Research in Mathematics and Data Science, Western Sydney University, Sydney, NSW, Australia; ^2^ Environmental Intervention Unit/Emerging Infections and Parasitology Laboratory, Infectious Disease Division, icddrb, Dhaka, Bangladesh; ^3^ Department of Biotechnology and Genetic Engineering, Noakhali Science and Technology University, Noakhali, Bangladesh; ^4^ Children’s Health and Environment Program, Child Health Research Centre, The University of Queensland, South Brisbane, QLD, Australia

**Keywords:** yogurt, gut health, Infant growth, randomized controlled clinical trial (RCT), LMIC (low and middle-income countries)

## Abstract

**Introduction:**

We evaluated the effects of yogurt supplementation and nutrition education to low educated mothers on infant-gut health at an early age.

**Methods:**

We designed a three-arm pilot randomized controlled trial with 162 infants aged 5-6 months and at risk of stunting (LAZ ≤-1 SD and >-2 SD at enrollment) living in slum areas in Dhaka, Bangladesh. Eligible children were randomized to receive, 1) nutrition education, 2) yogurt supplementation plus nutrition education or 3) usual care. Three faecal inflammatory biomarkers alpha-1 antitrypsin (AAT), myeloperoxidase (MPO), and neopterin (NEO) were measured before and after three months of yogurt feeding.

**Results:**

At the end of three months, there were no significant differences in the biomarker concentrations between the yogurt plus group and control. Compared to control, the adjusted mean faecal NEO concentration decreased by 21% (NEO: RR 0.79, 95% CI: 0.60, 1.04) and the adjusted mean faecal AAT concentration decreased by 8% (AAT: RR 0.92, 95% CI: 0.69, 1.22); whereas, the adjusted mean faecal MPO concentration increased by 14% (MPO: RR 1.14, 95% CI: 0.62, 2.09). Such changes were not apparent in the education only group.

**Discussion:**

After a three-month trial of daily yogurt feeding to children at risk of stunting and infant feeding education to their mothers, reduction in one inflammatory biomarker reached close to statistical significance, but not all of the measured biomarkers. The study did not finish its endline measurements at 6-month as designed due to COVID 19 pandemic. This has greatly impacted the interpretation of the results as we could not establish a decreasing trend in biomarker concentration with continued yogurt feeding.

## 1 Introduction

Millions of children worldwide are still experiencing life-threatening conditions due to malnutrition. In 2020, 149.2 million (22.0%) children under five years were stunted globally (UNICEF_WHO_WorldBankGroup, 2021). Half of the deaths of children under five result from stunting, an indicator of chronic malnutrition (UNICEF_WHO_WorldBankGroup, 2021). Until now malnutrition is widely prevalent in developing countries. Stunting prevalence in Bangladesh is pronounced, more than one in every four children is stunted (28%) (UNICEF, 2020). In addition to increasing morbidity and mortality, stunting causes the development of non-communicable diseases later in life (Eckhardt, 2006; Ezzati et al., 2018), adverse functional consequences like poor cognition and educational performance, low adult wage and low productivity (De Sanctis et al., 2021).

Inadequate dietary intake and repeated infections like diarrhoea are immediate causes of undernutrition, including stunting (United Nations Children’s F, 1991; Vaivada et al., 2020). An estimated six percent under-five deaths can be prevented by ensuring optimal complementary feeding (Black et al., 2003) since children can become stunted even with optimum breastfeeding, if adequate complementary feeding is not established from six months of age (Black et al., 2008; Badham, 2013). A systematic review by Dewey and Adu-Afarwuah (2008) estimated that education about child feeding practices alone was able to achieve a modest effect on weight (mean effect size 0.28) and linear growth (mean effect size 0.20), indicating that outcome can be further improved with nutritional component apart of education. Different nutrition interventions such as breastfeeding promotion, nutrition education, provision of complementary foods as well as vitamin and mineral supplementation (Vaivada et al., 2017; Dewey et al., 2021) revealed significant impact on improving indicators of child growth in low income countries (Imdad et al., 2011). While access to sufficient nutrition is the most crucial determinant, gut microbiota was also found to be associated with malnutrition (Smith et al., 2013; Million et al., 2017). The gut microbiota influences the somatotropic axis by regulating IGF-1 and growth hormone production affecting growth (Robertson et al., 2019). Also, growth faltering is possibly linked to gut microbiota as it exerts an influential role in inflammation and enteropathy (Robertson et al., 2019).

During the first few years of life rapid physical growth and cognitive development take place; in parallel, there is acquisition, selection and colonization of gut microbiota unless homeostasis is achieved (Wang et al., 2020). The first 1000 days of life, the period from conception to two years of age, is a crucial window for gut microbial maturation; any insult during this time has long term detrimental health impact. The trajectory of gut microbiota starts before birth; maternal nutrition and microbial composition, unsanitary conditions, diet, and repeated infections influence the evolution of microbial, metabolic and immunologic functions in children projecting long-term health outcomes like brain development and linear growth (Littlejohn and Finlay, 2021).

Microbial maturation could be impacted by various factors. Beyond six months, children are exposed to an increased number of pathogens from complementary foods, drinking water, soil, and their immediate environment (Robertson et al., 2019). Exposure to poor environmental sanitation leads to a subclinical condition called environmental enteric dysfunction (EED) characterized by increased intestinal permeability, decreased villous length, and microbial translocation (Keusch et al., 2014). In the low-income settings more than two-thirds of young children are affected by EED (Chaima, 2020). In these settings EED was identified to be associated with growth faltering. Research showed that intestinal inflammation was associated with subsequent acquired deficits in linear growth in infants (Kosek et al., 2013). The underlying mechanism through which EED influences growth is still under evaluation; one of the proposed pathways suggested that ingestion of enteric pathogens and toxins changes gut microbiota composition and their functions, this in turn leads to intestinal inflammation and derangement in its structural integrity (Chaima, 2020).

One of the key indicators of improved gut health is gut barrier function. There is considerable evidence that gut microbiota plays an important role in the development and maintenance of structural integrity of the intestinal barrier function in early infancy (Robertson et al., 2019). Probiotics are living organisms that alter the composition of the host microbial community through their immunomodulatory effects. Probiotics can influence gut barrier functions in various ways; these include controlling mucus production, preventing bacterial adhesion, enrichment of tight junctions, increasing cell survival, and inducing defense mechanisms (O’Flaherty et al., 2010; Bron et al., 2017). A systematic review by Heuven et al. looked at the effects of intervention that targeted gut-microbiota to improve child growth in low- and middle-income countries; five out of eleven studies demonstrated beneficial effects of probiotics on one or more growth parameters (Heuven et al., 2021). Systematic reviews indicated that undernourished children received more benefits from the probiotic intervention than well-nourished children (Onubi et al., 2015; Heuven et al., 2021).

Fermented foods have been an integral part of the human diet for thousands of years. Fermented foods are popular for their increased shelf life and for their unique changes in taste, flavor and functionality. Some of the most familiar fermented foods such as yogurt, kefir, or cheese contain viable organisms in significant quantities that promote gut health (Marco et al., 2017). The classic two yogurt bacteria associated with yogurt fermentation, namely, *Streptococcus thermophilus* and *Lactobacillus delbrueckii* subsp. *Bulgaricus*, can mostly survive human gastrointestinal transit (Mater et al., 2005; Morelli, 2014), which is important to exert their beneficial effects.

Not only for probiotic potential, fermentation is considered as a tool to enhance the nutritive value of foods by increasing availability of bioactive products and health-promoting end products like short-chain fatty acids (SCFA) (Annunziata et al., 2020). SCFA contributes in shaping the gut environment, modifying physiology of colon, act as an energy source of host cells and microbiota, they also take part in various host-signaling mechanisms (Ríos-Covián et al., 2016). Yogurt contains bioactive peptides that are produced due to hydrolysis of milk proteins by the lactic acid bacteria (Martínez-Augustin et al., 2014). A widespread health promoting properties have been attributable to the bioactive peptides such as anti-microbial, immune-modulatory, and mineral binding properties. Maintenance of intestinal barrier function is imminent for humans; different bio-active peptides have been proposed to play a role in altering intestinal barrier functions (Martínez-Augustin et al., 2014).

A number of epidemiological studies have examined the effects of fermented foods on health outcomes (Saran et al., 2002; He et al., 2005; Donovan and Rao, 2019) but none of the studies have explored the association between yogurt and growth of undernourished children at an early age of six month when children are at increased risk of exposure to pathogens affecting their gut health. Yogurt is a complementary food in various countries like Guatemala and Turkey (Köksal et al., 2015). Yogurt is a traditional fermented dessert item in Bangladesh produced at the household level (Hossain and Kabir, 2016); it should relatively be inexpensive compared to the probiotic supplements available in the market (Gordon, 2019).

A systematic review by Harper et al. considered intestinal inflammation as one of the five domains to describe the complex mechanism by which EED may contribute to stunting (Harper et al., 2018). As indicators of intestinal inflammation, alpha-1 antitrypsin (AAT), myeloperoxidase (MPO), and neopterin (NEO) are frequently used along with other indicators like regeneration gene 1 Beta (Reg 1B) or calprotectin levels (Mahfuz et al., 2017; Morais and Silva, 2019). Large longitudinal trials such as SHINE trial in Zimbabwe (Prendergast et al., 2015) and WASH,B study in Bangladesh (Lin et al., 2020) have tested these biomarkers as a measure of intestinal inflammation along with tests for other domains. In this pilot trial, we choose to examine only these three biomarkers since they are stable, easy to process, and feasible to test within the available budget. Thus, the objective of this study was to investigate if provision of daily yogurt supplementation to 6-month old infants at risk of stunting could improve their gut health by reducing intestinal inflammation over a three months period. We measured three faecal inflammatory biomarkers alpha-1 antitrypsin (AAT), myeloperoxidase (MPO), and neopterin (NEO) before and after the yogurt intervention as indicators of intestinal inflammation to evaluate the impact of the intervention.

## 2 Methods

### 2.1 Study design

**Figure 1:**
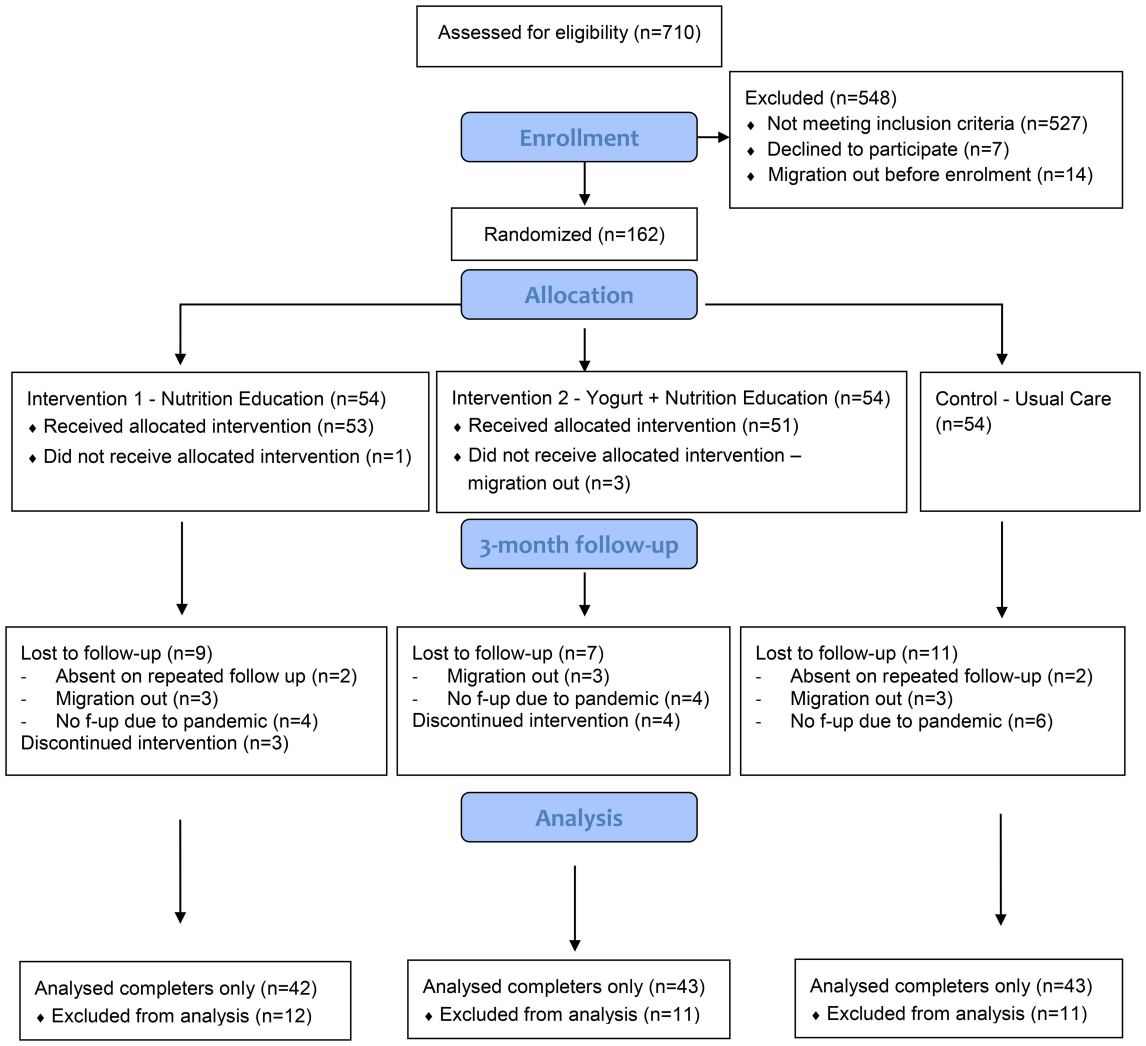
Study flow diagram.

This study was a three arms pilot randomized controlled trial conducted in low-income communities from five areas: *Mohakhali, Nakhalpara, Korail, Badda*, and *Kalshi* located at the central part of the Dhaka city corporation area. Considering the nature of the study activities (daily distribution of yogurt), we choose to limit the study sites within a reasonable distance from the research center for everyday commute using public transport. The general environmental and housing condition, water supply and sanitation systems are similar across the urban slums (Dana, 2011; Soma et al., 2022); the selected five study locations are therefore representative of typical urban slums. Participants were randomly assigned to a) nutrition education, b) yogurt supplementation plus nutrition education, or c) the usual-care (control) groups (**Figure 1**). Adequate nutrition is a prerequisite for better growth and nutrition education is the minimal essential step to improve child feeding practices, particularly for low educated mothers. We therefore added an education-only group to isolate the effect of yogurt supplementation from nutrition education when both groups were compared to the usual care group. The study was designed for six months with two follow up assessments; however, due to the COVID-19 pandemic, data collection had to stop midway with one follow up assessment only.

### 2.2 Ethical approval and trial registration

The study received ethical approval from the Ethical Review Committee of the International Centre for Diarrhoeal Disease Research, Bangladesh (icddr,b) (PR-19062) and the Human Research Ethics Committee at the Western Sydney University (H12719). The trial was registered at *ClinicalTrials.gov* with a trial registration number NCT04067284.

### 2.3 Study sites and participants

Infants aged 4-6 months and at risk of stunting at enrollment (length-for-age z-score LAZ ≤-1 SD to >-2 SD), and their mothers’ education reported to be less than ten years were eligible for the study. Children with moderate to severe malnutrition, congenital abnormalities or any chronic conditions at screening were not included in the study. After identifying the children from the vaccination database at the Expanded Program of Immunization (EPI) Center, Mohakhali, Dhaka, the field research assistants (FRAs) visited households to complete the screening process to determine eligibility. The FRAs provided participant information sheets to the households with eligible participants. If needed, the FRAs read the information sheet to the participants; they answered all queries and concerns, and obtained the informed written consent from the fathers of the index children as household heads.

### 2.4 Randomization, concealment and blinding

We have done block randomization with a block size of four, using a predetermined randomization sheet generated by a co-investigator at Western Sydney University. We allocated participants into any three study groups in equal proportion. Allocation was concealed to the research staff until the baseline assessment was completed. However, since the intervention included visible components, FRAs were not blinded for the subsequent anthropometric and dietary assessments, neither were the participants. Allocation was also concealed for the faecal sample testing for biomarkers.

### 2.5 Intervention plan

#### 2.5.1 Intervention-1: Nutrition education (education-only group)

This group received monthly nutrition education sessions at the household level. We adapted behavioral recommendations from previous promising interventions on Bangladeshi infants namely the WASH Benefits Bangladesh trial (Luby et al., 2018). Illustrated flip chart was used to deliver the messages to the mothers and household members. In addition to flip charts, each session included quizzes, flashcard games or storytelling components. Messages emphasized on continued breastfeeding, dietary diversity in complementary feeding, safe water, and handwashing with soap before feeding. In addition to the conventional methods used for nutrition promotion, we introduced a unique tool for self-monitoring mother’s feeding practices. Mothers received a pictorial calendar with colored stickers. Each color represented a different food group: yellow for energy yielding foods, red for body building foods, and green for protective foods. Mothers were asked to fill in the calendar each day according to what food groups they have fed to their children.

#### 2.5.2 Intervention-2: yogurt supplementation + nutrition education (yogurt plus group)

This group received the nutrition education mentioned above at the same frequency and a cup of yogurt (50 gm) every day for the index children. To minimize the supply of additional calories from yogurt, minimum serving size recommended by the nutritionists was delivered to the children ([]Probio (For kids)).

##### 2.5.2.1 Yogurt making, distribution, and quality control

The Codex standard defined yogurt as the product of milk fermentation by *Lactobacillus delbrueckii* subspecies *bulgaricus* and *Streptococcus thermophilus* (Marette and Picard-Deland, 2014). Following this definition, we made yogurt every day in the kitchenette of the research unit at the icddr,b. The FRAs made yogurt from cow’s milk as full cream milk powder and yogurt culture (**Supplementary Figure 1**). A commonly available full cream milk powder was selected, and yogurt culture was imported from Australia manufactured by Green Living Australia. Classic yogurt bacterial strains: *Lactobacillus bulgaricus* and *Streptococcus thermophilus* were present in the highly concentrated powder yogurt culture. Milk powder was reconstituted as instructed on its packaging and inoculated with yogurt culture at the proportion recommended by the manufacturer. As more children were enrolled in the study, we standardized our method of preparing a greater amount of yogurt. No sugar or flavor was added to the yogurt. Necessary hygienic conditions were maintained at each step of yogurt making. The yogurt was incubated overnight (12-14 hours) at room temperature which was around 30 degrees centigrade most of the months of yogurt making (October to March). To ensure a constant warm environment required for incubation, yogurt cups were placed in airtight plastic boxes and were wrapped with blankets. The room where the yogurt cups were left for incubation had no windows or doors open, no air-conditioning was running. In the morning, the batch of yogurt was transferred to a refrigerator with a temperature of 3-5 degrees centigrade. Yogurt was distributed to the households in disposable cups along with spoons using cooler boxes. Temperature within the cooler was monitored by thermometer, which was maintained between 4-10 degrees centigrade throughout the distribution period. Any leftover yogurt was discarded, and a fresh batch of yogurt was made on the next day. The basic principles of yogurt making, and detailed steps are demonstrated in the following link by the manufacturer, Green Living Australia (https://www.greenlivingaustralia.com.au/mild-dairy-yoghurt-culture).

We tested the manufactured yogurt at the Laboratory of Food Safety and One Health at icddr,b twice a week for identification and enumeration of the yogurt culture bacteria using selective plating methodologies for the mixed culture of *Lactobacillus bulgaricus* and *Streptococcus thermophilus* as described in earlier research (Tabasco et al., 2007). Yogurt bacteria are sensitive and degrade during processing and while passing through the stomach acid. Generally, 1-100 million CFU of viable organisms per gram of food is recommended for administration (Fernandez and Marette, 2017). The average concentration of culture bacteria measured in our prepared yogurt was nearly 100 million CFU/g of yogurt (2.6x10^8^ CFU/g for *Streptococcus thermophilus* and 1.1x10^8^ CFU/g for *Lactobacillus delbrueckii* subsp. *Bulgaricus)*.

#### 2.5.3 Usual care (control group)

This group received no intervention from the research team and represented the current recommended practices among healthcare providers. On demand primary health care services are provided to the mothers and children by the local government, nonprofit organizations (NGO), or private sector facilities (Adams et al., 2015).

### 2.6 Outcome measures and covariates

Primary outcome: The primary outcome of this study was the change in mean concentration of three selected faecal biomarkers at 3-month follow up. Presently, there are no definite diagnostic criteria for EED (Harper et al., 2018). Confirmed diagnosis of EED requires intestinal biopsy, which is an invasive and expensive procedure; metagenomic sequencing is also a better measure to assess the changes in microbial community but was not feasible to perform in Bangladesh.

Intestinal inflammation can cause extravasations of AAT due to mucosal ulceration and impaired permeability. It can be detected in faecal samples since AAT is one of the principle serum proteins that can resist intestinal proteolysis and thus excreted in feces in intact form (Kosek et al., 2013). AAT is the classic faecal marker of protein losing enteropathies. Neopterin is an indicator of immune activation, when T-lymphocytes are activated they stimulate macrophage and dendritic cells to produce neopterin (Kosek et al., 2013). Myeloperoxidase indicates neutrophil activities in the gut mucosa, it is found to be associated with disease activities in inflammatory bowel diseases (Kosek et al., 2013). Cut-off values for most of these biomarkers have not been established in children with malnutrition (Keddy et al., 2021), thus any reduction in concentration was considered to be beneficial as an indicator of improvement in gut health. Another primary outcome of the study was change in mean length-for-age z-score (LAZ) from baseline to 3-month follow-up across arms (main outcome manuscript is under review).

Covariates: Most of the alleged confounders such as cesarean section delivery, breastfeeding status, mother’s education, and household water-sanitation facilities were measured at baseline. Infant’s dietary pattern was assessed using the WHO infant food frequency questionnaire (FFQ) at baseline and 3-month follow up. Primary caregiver, which was mostly mothers, completed this 24-hour recall. Two modules on household food insecurity and household wealth assessment, adapted from the WASH Benefits study (Luby et al., 2018), were completed during the 3-month follow-up to optimize the time required for each assessment round. Considering the crowded slum environment and time constraints of the mothers, assessment rounds required to be quick and efficient.

History of illness: we collected information on a child’s illness episodes and exposure to antibiotics during the trial, given the associations between these exposures with gut health and children’s potential growth. We used a previously validated structured questionnaire from the WASH,B study (Luby et al., 2018). Every two weeks the FRAs visited the households to ask mothers to recall if the index child experienced any fever, cough, runny nose, wheeze, diarrhoea or loose stool in the last 14 days. If yes, additionally they were asked if they had taken any medication for those symptoms.

#### 2.6.1 Sample collection

Stool sample collection was conducted from all infants within 14 days of baseline (before the intervention was launched) and at 3-month follow up assessment. Stool collection required at least two visits to each household. The day before a sample was collected, the FRAs delivered a stool collection kit to the mothers and briefed them to collect stool samples from their children on the following morning. Mothers collected 10 ml of stool from a diaper or clothing using a plastic scoop (integrated into the supplied storage container). A stool sample was not allowed to be collected from the floor; diarrhoeal stool was also not collected as a specimen. After delivering the sample collection kits, FRAs supervised the mother to collect the stool sample as soon as possible; FRAs carried the samples to the lab using cool boxes maintaining a temperature of 4°C to 10°C. The collected stools were allocated to 2.0ml microcentrifuge tubes within four hours of collection. The aliquots were primarily stored in a-20°C freezer and then transferred to a central bio-repository at -80°C every 7-10 days until the biomarkers were measured.

#### 2.6.2 Laboratory procedure

The laboratory assays were performed in the Emerging Infections and Parasitology laboratory at icddr,b in Dhaka, Bangladesh. A total of 284 stool samples were tested blindly using commercially available Enzyme-Linked Immunosorbent Assay (ELISA) kits for measuring human Myeloperoxidase (MPO) (Immundiagnostic AG, Germany, Cat. KR6630), Neopterine (NEO) (GeneWay Biotech Inc, San Diego, CA, Cat. 40-371-25012) and Alpha-1-Antitrypsin (A1AT) (Immuchrom, Biovendor, Chandler, NC, Cat. RIC6200). The assays were performed and optimized according to an earlier published MAL-ED study (Kosek et al., 2013). The kit’s performance was checked using known samples before starting the study sample analysis. Baseline samples that lacked a follow up measurement were excluded from this analysis (N=30).

### 2.7 Monitoring compliance

The FRAs tried to remain present when yogurt was fed to the children. If it was left for a later feeding, mothers were asked to feed it within two hours of delivery; FRAs confirmed feeding with a follow up phone call to the mothers. The amount of daily consumption was recorded in a logbook. Adherence to yogurt intervention was dichotomized to more than 80% or below; that is children who consumed at least half cup (25 gm) of yogurt on 80% or more of the days the yogurt was supplied were considered to be adherent to the intervention.

### 2.8 Sample size and data analysis

The sample size of the trial was calculated based on the ability to detect a mean difference of 0.3 in LAZ between intervention groups compared to control after 6 months when children reach 12 months of age. We estimated 120 children will be needed to detect this difference at a 0.05 precision with 80% power. Allowing for 25% loss to be followed we adjusted the required sample to 162 children. We randomized 162 children to three study groups at an equal proportion. This study refers to 127 infants with no missing data on stool and growth parameters at baseline and 3-month follow-up, when the study had to stop due to lockdown for COVID-19 pandemic. For the biomarker study, we conducted a *post-hoc* power calculation to detect significant changes in biomarkers between any intervention groups against control based on complete cases of stool data. This calculation was based on the mean baseline biomarkers levels of the control. Assuming no changes in the control we were able to detect a minimum reduction in concentration of ≥33% AAT, ≥62% for MPO, and ≥31% for NEO for a sample size of 43 in control and 42 in any treatment arm keeping 80% and p-value of 0.05.

As appropriate, we compared demographic and other baseline covariates between groups using t-tests and chi-square tests. Infant food frequency data was analyzed according to WHO recommended Infant and Young Child Feeding (IYCF) indicators (WHO, 2021). All three biomarker concentrations were highly skewed, we log transformed the biomarker concentrations to make their distribution near normal, and minimize the effect of outliers. Mean log concentration of biomarkers at 3-month follow up was compared across groups using ANCOVA, adjusting for baseline biomarker values. In case of significant or near significant between -groups differences we also measured the Cohen’s d effect size on the log transform data. This formula is appropriate when the group has similar SDs and same sample size (Cohen, 2013). We checked association of fecal biomarkers and potential confounding factors such as cesarean section delivery, diarrhoeal diseases, antibiotic intake and dietary diversity, none of them were statistically significant. To minimize any residual confounding effects, we included these variables in the model. The model that addressed maximum variability was selected as the final model to estimate changes in biomarker concentration. The final model included diarrhoea, cough, antibiotic intake, and minimum dietary diversity score as covariates.

ANCOVA was also used to see the association between anthropometric indices (LAZ and WAZ) and log biomarker concentrations at follow up expressed as response ratio (RR) and adjusted for baseline LAZ and WAZ indices. We divided the concentrations into three categories: <25 percentile, 25-75 percentile, and>75 percentile and looked at their relationship with linear growth considering <25 percentile as the reference group. A Principal Component Analysis (PCA) based on the natural log of the three biomarkers at follow up was performed to measure a composite score of environmental enteropathy. The composite index was then divided into tertiles to look at their graded relationship with linear growth (Kosek et al., 2013).

## 3 Results

The mean age of the children during baseline assessment was 180 days (95% CI: 179, 181); gender was balanced across the study groups (**Table 1**). The mean duration of mother’s education was 4.6 years (95% CI: 4.1, 5.1). Birth through cesarean section was more common among the education only group compared to the yogurt plus group and control (education only 50.0%, yogurt plus 35.7%, control 30.2%; p-value 0.16). All of the households had improved but shared latrine facilities. Almost all households received water supply from the city corporation. More households (not significant) in the yogurt plus group were in the richest wealth quintile compared to education only or control groups (education only 11.9%, yogurt plus 31.0%, control 16.3%; p-value 0.33) (**Table 1**).

**Table 1:** Demographic characteristics of the study children and households.

Indicators		Control N=43	Education N=42	Yogurt + education N=42	*p*-value
Child age in days (mean, 95% CI)		180 (178, 182)	180 (179, 181)	180 (179, 181)	1.00
Child gender (male)		51.2	54.8	50.0	1.00
Type of delivery (cesarean section)		30.2	50.0	35.7	0.16
Exclusive breastfeeding for first 6 months		30.2	31.0	23.8	0.73
Continued breastfeeding		100.0	95.2	92.9	0.23
Mother’s education (mean, 95% CI)		4.4 (3.5, 5.2)	4.9 (4.1, 5.6)	4.6 (3.8, 5.5)	0.72
Shared improved sanitation facilities (pour-flush latrine to piped sewer system)	Shared with <10 households Shared with ≥10 households	72.1 27.9	64.3 35.7	69.1 31.0	0.74
Sources of drinking water (municipal supply)		97.7	97.6	100.0	1.00
Household wealth	Quintile 1 (poorest)	20.9	23.8	16.7	0.33
	Quintile 2	27.9	21.4	9.5	
	Quintile 3	16.3	21.4	23.8	
	Quintile 4	18.6	21.4	19.1	
	Quintile 5	16.3	11.9	31.0	
Prevalence of food insecurity	Food secure	51.2	40.5	54.8	0.18
	Mildly food insecure	9.3	7.1	7.1	
	Moderately food insecure	28.0	31.0	31.0	
	Severely food insecure	11.6	21.4	7.1	

Mothers’ reported illness episodes within the 3-month study period were not different across groups (**Table 2**). The most frequently reported symptom was cough with an average of more than three episodes in three months. The reported diarrhoeal illness was less than one episode in the 3 month period. Antibiotic intake was also similar across groups (average 1.5 occasions in three months) (**Table 2**). Adherence to the yogurt consumption was 86% and no adverse event was associated with yogurt consumption. Yellow fruits and vegetables consumption was significantly higher in the yogurt plus group compared to control. No significant difference was seen in the proportion of consumption of any other food groups in the past 24-hours between the two intervention and control groups (**Supplementary Table 1**).

**Table 2:** Study activities across study groups.

Indicators	Control N=43	Education N=42	Yogurt + education N=42	*p*-value
	Mean (95% CI)	Mean (95% CI)	Mean (95% CI)	
Average duration of study (days)	91.0(89.9, 92.1)	92.5 (90.2, 94.8)	90.7 (90.4, 91.0)	0.19
Average number of illness tracking visits within 3 months	8.3 (7.8, 8.8)	8.8 (8.2, 9.3)	9.0 (8.5, 9.5)	0.13
Average number of episodes of illness within 3 months				
Fever	3.2 (2.7, 3.7)	3.4 (2.8, 4.0)	3.1 (2.5, 3.8)	0.81
Diarrhea	0.4 (0.2, 0.6)	0.7 (0.4, 1.0)	0.4 (0.2, 0.6)	0.11
Cough	3.6 (2.9, 4.3)	3.6 (3.0, 4.3)	3.6 (2.9, 4.4)	1.00
Wheezing	2.2 (1.7, 2.7)	1.9 (1.4, 2.5)	2.6 (2.0, 3.2)	0.26
Average number of occasions of antibiotic intake within 3 months	1.6 (1.2, 2.0)	1.4 (1.0, 1.8)	1.5 (1.1, 2.0)	0.87
Average duration of yogurt delivery (days)	0	0	80.6 (77.6, 83.5)	–
Average proportion of days of yogurt intake	0	0	86.1 (80.6, 91.6)	–

**Table 3** reports the baseline and follow-up biomarker concentrations as geometric means and the effect of the interventions compared to control after adjustment for episodes of diarrhoea, cough, antibiotic intake and minimum dietary diversity score. There were no differences in the mean concentration of AAT, MPO and NEO between the study groups at baseline. For the whole study population, the geometric mean concentration of AAT was 465 µg/g, the mean MPO was 7,780 ng/ml, and the NEO was 1726 nmol/L at baseline.

**Table 3:** Effect of nutrition education and daily yogurt supplementation for three months on faecal biomarkers of inflammation among children aged 6-9 months in Dhaka’s slum.

Complete case analysis N=127					
	Baseline Geometric Mean	3-month follow up Geometric Mean	^‡^RR	95% CI	*p*-value
Log alpha 1 antitrypsin (AAT) µg/g					
All	465	446		–	–
Control	447	454	1.00	–	–
Education	434	445	1.06	0.80, 1.41	0.69
Yogurt + education	518	438	0.92	0.69, 1.22	0.57
Log myeloperoxidase (MPO) ng/ml					
All	7,780	10,020			
Control	8,854	8,782	1.00	–	–
Education	5,372	10,913	1.20	0.64, 2.22	0.57
Yogurt + education	9,872	10,531	1.14	0.62, 2.09	0.67
Log neopterin (NEO) nmol/L					
All	1,726	1,503			
Control	1,883	1,678	1.00	–	–
Education	1,677	1,496	0.88	0.67, 1.17	0.38
Yogurt + education	1,624	1,348	0.79	0.60, 1.04	0.09

^‡^Response Ratio (RR) was calculated using ANCOVA adjusted for baseline value of the biomarkers, diarrhoea, cough, antibiotic consumption, and minimum dietary diversity score.

There was no significant difference in biomarker concentration among the yogurt plus group compared to control after 3-month of intervention. However, there was a tendency of decline in faecal NEO concentrations only in the yogurt plus group, although the Cohen’s d effect size compared to the control group was small (0.36, 95%CI -0.07, 0.79). The adjusted mean faecal NEO concentration was 21% lower among the yogurt plus group compared to control (NEO: RR 0.79, 95%CI: 0.60, 1.04, p=0.09) (**Table 3**).

**Figure 2:**
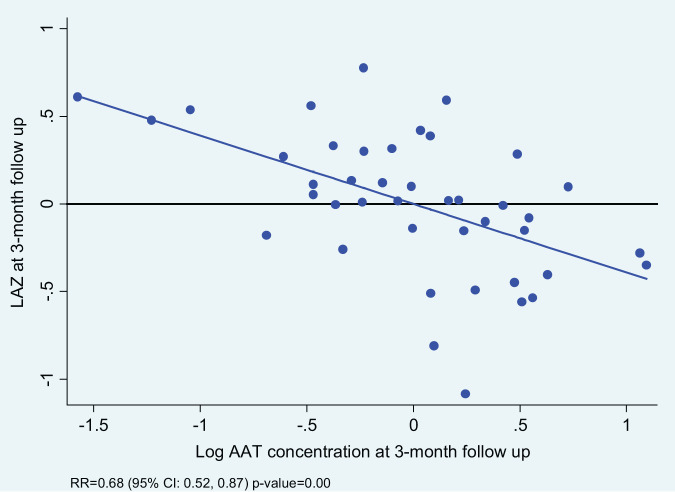
Relationship between log-AAT concentration and length-for-age z-score (LAZ) at 3-month follow among yogurt + education group.

**Figure 3:**
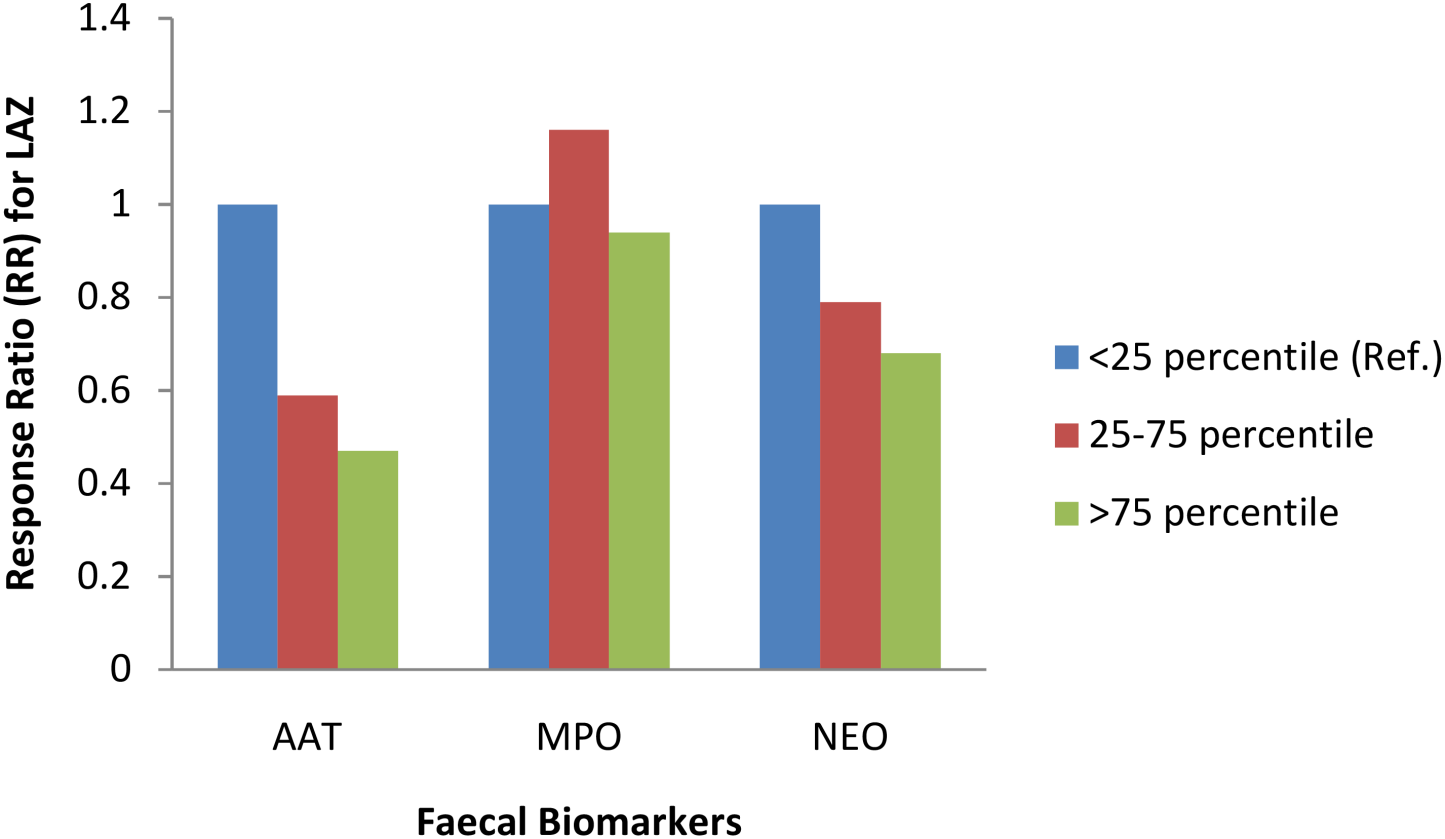
Association between three faecal biomarkers of inflammation and length-for-age z-score (LAZ) at 3-month follow up among yogurt + education group. ^‡^Response Ratio (RR) was calculated using ANCOVA adjusted for baseline value of LAZ, child gender, household wealth, fever, diarrhoea, acute respiratory infections, antibiotic consumption, number of illness tracking visits, exclusive breastfeeding status, mode of delivery, mother’s education, and minimum dietary diversity score.

For the whole study population at 3-month follow up, when log concentration of biomarkers increased linear growth decreased. This means that increase in biomarker concentration was associated with a shift of LAZ away from the reference population mean (LAZ=0), albeit these negative shifts were not statistically significant. In a stratified analysis by study groups, education only and control groups showed heterogeneous relationship between biomarkers and linear growth, with the exception of the yogurt plus group, where increase in all three biomarker concentrations were consistently associated with decreased linear growth (**Table 4**). In the yogurt plus group, one unit increase of log concentration of AAT was associated with 32% decrease in mean LAZ (RR 0.68, 95%CI: 0.52, 0.87) or in other words, one unit decrease in log concentration of AAT was associated with 47% increase in mean LAZ, which demonstrated by shift in mean LAZ toward population mean and this shift was statistically significant (**Table 4**). This relationship became intensified when low complaint children were excluded from the analysis (RR 0.64, 95%CI: 0.50, 0.81). **Figure 2** demonstrates the inverse relationship between AAT concentration and LAZ at three month follow up. When log concentration of AAT was categorized into percentiles, in the yogurt plus group, children in the highest log AAT concentration category had the lowest mean LAZ (i.e., getting away from the population mean) compared to mean LAZ in the lowest log concentration category (mean LAZ in <25 percentile vs 25-75 percentile: RR 0.59, 95% CI 0.37, 0.94; >75 percentile: RR 0.47, 95% CI 0.28, 0.81) (**Figure 3**; **Supplementary Table 2 and 2A**).

**Table 4:** Association of child growth parameters and faecal biomarkers of inflammation at 3-month follow up among study children aged 9 months in Dhaka’s slum.

	LAZ at 3-month			WAZ at 3-month		
	^‡^RR	95% CI	*p*-value	^‡^RR	95% CI	*p*-value
All groups N=127						
Log AAT concentration at 3-month	0.89	0.76, 1.05	0.16	0.90	0.81, 1.02	0.09
Log MPO concentration at 3-month	0.96	0.89, 1.04	0.37	0.98	0.93, 1.04	0.55
Log NEO concentration at 3-month	0.91	0.77, 1.09	0.31	1.05	0.92, 1.19	0.46
Control N=43						
Log AAT concentration at 3-month	1.02	0.69, 1.52	0.90	1.00	0.73, 1.37	0.99
Log MPO concentration at 3-month	0.95	0.76, 1.20	0.68	0.91	0.75, 1.09	0.29
Log NEO concentration at 3-month	0.82	0.47, 1.44	0.48	1.27	0.82, 1.97	0.28
Education N=42						
Log AAT concentration at 3-month	1.04	0.82, 1.32	0.75	0.98	0.78, 1.22	0.84
Log MPO concentration at 3-month	1.02	0.90, 1.17	0.70	1.00	0.89, 1.12	0.96
Log NEO concentration at 3-month	1.28	0.96, 1.70	0.09	1.14	0.89, 1.47	0.29
Yogurt + education N=42						
Log AAT concentration at 3-month	0.68	0.52, 0.87	<0.01	0.83	0.66, 1.04	0.09
Log MPO concentration at 3-month	0.98	0.85, 1.13	0.74	1.00	0.90, 1.13	0.91
Log NEO concentration at 3-month	0.81	0.57, 1.15	0.23	0.91	0.67, 1.25	0.55

^‡^Response Ratio (RR) was calculated using ANCOVA adjusted for baseline values of growth parameters, child gender, household wealth, fever, diarrhoea, acute respiratory infections, antibiotic consumption, number of illness tracking visits, exclusive breastfeeding status, mode of delivery, mother’s education, and minimum dietary diversity score

We found that children who had 80% or more compliance with the yogurt intervention, had a notable reduction in log AAT concentration at follow up even though not statistically significant (RR 0.63, 95% CI 0.38, 1.03) compared to those who had a compliance of less than 80% (**Supplementary Table 3**).

## 4 Discussion

After a three-month intervention of daily supplementation of yogurt to infants at risk of stunting along with infant feeding education to mothers having low educational attainment, no significant decrease was seen in the inflammatory faecal biomarker concentrations. While faecal NEO concentration showed a tendency to decline, changes in AAT and MPO concentrations were equivocal in the yogurt plus group compared to control. In the yogurt plus group, increase in fecal AAT concentration was associated with significant decrease in linear growth measured by LAZ. Infants with high compliance with the yogurt intervention showed considerable decrease in faecal AAT concentration compared to the infants with low compliance.

Improved intestinal microbiota is a plausible newer strategy to prevent undernutrition among children. Research suggests an association between gut microbiota and child malnutrition; it is recommended that future interventions should target to support a healthy gut microbiota (Iddrisu et al., 2021). There is paucity of trials that measured fecal inflammatory biomarkers among infants following intervention with fermented foods; however, there are studies with probiotics and/or prebiotics supplements (Viljanen et al., 2005; Oswari et al., 2013; Castanet et al., 2020). Castanet et al. investigated the effects of different feeding regimens on early gut maturation in healthy full term infants from three European countries enrolled at birth. The supplement containing formula lactoferrin, probiotic and prebiotic (bovine milk derived oligosaccharides, BMOS) favored gut maturation compared to control. Children in this group got closer to the breastfed infants for some of the measured biomarkers such as AAT (Castanet et al., 2020). Measurement of growth outcome is relatively more common in microbiota-targeted nutritional interventions, even though not as a primary outcome oftentimes. Studies using short term microbiota-targeted nutritional interventions (around 3-months) on healthy or malnourished under five children have shown increase in body weight (Surono et al., 2011; Kara et al., 2019; Kusumo et al., 2019; Mai et al., 2021); whereas, studies with longer duration of intervention (six months or more) documented significant improvement in linear growth (Saran et al., 2002; He et al., 2005). Our study achieved minimal duration (3-months) that is necessary for measurable effects on growth (Heuven et al., 2021), which might be one of the reasons it failed to show significant results.

Change in gut microbiota in EED could result in change in fecal inflammatory biomarker concentration among children. A recent review by Keddy et al. highlighted further investigation on the potential of diagnostic use of biomarkers for EED and child malnutrition (Keddy et al., 2021). The MAL-ED study, one of the few studies that looked at the biomarkers of infection in low- and middle-income countries (LMICs), pointed out that more extensive biomarker screening and follow up studies are required to determine promising biomarker candidates (McCormick et al., 2017). Only a limited number of data is presently available which is not sufficient to direct development of new interventions (Keddy et al., 2021). Thus, precise estimation of effectiveness of the yogurt intervention was not possible due to absence of reference cut-off values of the measured inflammatory biomarkers. However, improvement in gut health could be anticipated if biomarkers that are identified to have significant association with enteric inflammation drop.

All three biomarkers had high mean concentration across study groups. By comparison, children at that age living in high-income countries reported much lower means for AAT 270 µg/g, MPO 2,000 ng/ml, and NEO 70 nmol/L (McCormick et al., 2017). The biomarker concentrations were also higher than that was reported in the MAL-ED study investigating children from the LMICs (McCormick et al., 2017). The markedly high concentration of biomarkers at baseline, when the children were 6 months of age and mostly did not start complementary feeding, indicated that severe intestinal inflammation was initiated very early in life among the study children living in slums.

One of the key determinants of microbial composition and diversity is dietary intake. Research indicated that long-term dietary patterns, even short-term dietary interventions, are linked to the composition and diversity of the gut microbiome (Donovan, 2017). The fermentation-associated microbes present in fermented foods are proposed to direct the health benefits they confer. Likewise, yogurt starter cultures *Streptococcus thermophilus* and *Lactobacillus delbrueckii* subsp *bulgaricus* showed probiotics properties when consumed (Kok and Hutkins, 2018). The literature suggests several mechanisms through which fermented foods exert their health benefits to host; potential probiotics effects of constituent microorganisms, fermentation derived bioactive compounds, and reduction of anti-nutrients (Dimidi et al., 2019).

In this study, we observed a slight reduction in faecal NEO concentrations in the yogurt plus group at 3-month follow up compared to the control group. One of the challenges in explaining our findings is our inability to attribute this reduction solely to yogurt consumption or to improvement of consumption of certain food groups that had an impact on gut health, or to a combination of both. We recorded a tendency of increased consumption of certain food groups (flesh foods, yellow fruits and vegetables) among the yogurt plus group as well as in the education only group, albeit to a lesser extent. Yet, we did not see any pattern in the biomarker concentration among the education only group, which would be expected if ‘other food groups’ explain the results. We adjusted dietary diversity score in the final model for estimating changes in biomarker concentration; in addition, our model was adjusted for differences in other potential confounding factors that might impact gut health at early age such as cesarean section delivery, diarrhoeal diseases, and antibiotic intake along the trial period.

The association between these three fecal biomarkers and growth parameters is inconsistent. A cohort study in Bangladesh by Naylor et al. found MPO was negatively associated with HAZ and WAZ from enrollment to one year follow-up (Naylor et al., 2015). A case control study with Egyptian children has indicated that children with malnutrition were more likely to have higher AAT and NEO biomarkers than non-malnourished children (El Wakeel et al., 2021). On the other hand, in young Tanzania children there was no association between these biomarkers and child growth indicators (Wirth et al., 2019). Therefore, we wished to examine whether any of these biomarkers was associated with growth parameters in any of the study groups. Compared to control, the yogurt plus group showed significant inverse relationship between LAZ and AAT concentration. A substantial decrease in AAT concentration was observed among the high compliant children compared to the low compliant children, although the result was significant at 10% level only and was not significant with the other two biomarkers. Intestinal barrier integrity is an endorsement of gut health; impairment of intestinal barrier function can result in a condition of chronic immune activation that diverts nutrients to fight infection rather than growth (Mbuya and Humphrey, 2016). Our results were not conclusive to support if yogurt culture bacteria or bioactive peptides present in yogurt have had played a role in maintenance, reinforcement, and repairment of the intestinal barrier functions (Martínez-Augustin et al., 2014).

A longitudinal study of 72 Gambian children that were followed for 13 months found that long term height and weight gains were inversely associated with mean faecal NEO concentration (Campbell et al., 2004). In our study, a similar inverse relationship was found between NEO categories and length in the whole study population at a significance level of 10%. Yogurt plus intervention showed an increasing inverse correlation, meaning that as the NEO concentration shifted from moderate to high, LAZ worsened a step further, but this was not statistically significant. The MPO concentration showed no association with length. This might be due to its highly skewed distribution, which was not fully normalized even after log transformation. However, to keep the analysis consistent and easier to interpret we continued with the log transformation.

We found no consistent pattern of association between faecal inflammatory biomarkers and weight gain or mean WAZ across study groups. Yogurt can improve the diet quality of children by providing a significant amount of macro and micronutrients. Keeping this in mind, we supplied a minimum amount of yogurt that served the recommended dose of viable organisms but only 5% of the daily calorie requirements of the infants if the full supplied amount was consumed (Abeshu et al., 2016). Yogurt consumption was suggested to be inversely associated with weight gain or obesity, that is more yogurt consumption was associated with less weight gain or obesity in most of the studies done previously (Marette and Picard-Deland, 2014). One of the proposed mechanisms behind this health benefit is the probiotics potential of yogurt that might have beneficial effects on gut health (Marette and Picard-Deland, 2014). Yogurt nutrients contribute to healthy growth; yogurt may also help maintain a negative energy balance and thus lead to a healthy body weight (Marette and Picard-Deland, 2014).

Strength and limitations

This pilot study was designed for shorter duration with minimum sample size. As a result of COVID 19 pandemic, the study duration was cut even shorter. This has greatly impacted the interpretation as we could not establish a decreasing trend in biomarker concentration with continued yogurt feeding. We measured three simple and relatively low cost faecal biomarkers, whereas genome sequencing might have been more appropriate to see the changes in the microbial community following yogurt consumption. An ideal way to answer the study question might be to have a milk comparison arm (i.e. compare yogurt made from cow’s milk with cow’s milk). We wanted to introduce the yogurt supplementation as soon as the infants start complementary feeding, anticipating that the infant gut microbiome has undergone minimum assault; cow’s milk is not recommended for children less than 12 months. Another reason for taking younger children was that during 6-12 months of age a measurable change in growth is expected. To keep the study activities within the available fund, we had to omit laboratory testing of the faecal coliform bacteria to exclude food contamination, which are limitations of the study.

However, we have performed a blinded testing of the biomarkers in the highly specialized laboratory at icddr,b that had experience conducting such tests in large longitudinal studies (Kosek et al., 2013). Biomarkers are objective measures, free from potential reporting bias and thus imply more validity to the results. Blinded analysis of the stool samples was corroborated with the anthropometric measurements that revealed thought-provoking findings. We designed a comprehensive intervention to target important determinants of child nutrition adapted from previous successful interventions like emphasizing adequate nutrition, safe water and hygiene. We focused on improving gut health and increasing compliance of child feeding practices of low educated mothers through self-monitoring using pictorial calendar. In addition, this study has application in the real world; yogurt should be a daily food prepared in the household without pathogen testing. We found our yogurt was safe for the children and therefore such intervention is scalable.

## 5 Conclusion

After a three-month trial of daily yogurt feeding to children at risk of stunting and infant feeding education to their mothers, reduction in one inflammatory biomarker reached close to statistical significance, but not all of the measured biomarkers. We designed this pilot clinical trial with optimum sample size and indirect outcome measures to test a novel hypothesis; the study did not finish its endline measurements at 6-month as designed due to the COVID 19 pandemic. This has greatly impacted the interpretation of the results as we could not establish a decreasing trend in biomarker concentration with continued yogurt feeding. We recommend further investigation of this meaningful and affordable intervention with children living in low-income settings since fermented foods, particularly yogurt, have wide range of beneficial impact on gut health.

## Data availability statement

The original contributions presented in the study are included in the article/**Supplementary Material**. Further inquiries can be directed to the corresponding author.

## Ethics statement

The studies involving human participants were reviewed and approved by Ethical Review Committee, icddr,b and Human Research Ethics Committee, Western Sydney University. Written informed consent to participate in this study was provided by the participants’ legal guardian/next of kin.

## Author contributions

KJ drafted the manuscript under the guidance of DM and input from all listed co-authors. KJ drafted the research protocol under the guidance of DM and KA; she coordinated input from the study team through-out the project. KJ, SP, and MR oversaw piloting and subsequent study implementation, contributed to refinements in interventions and measurements and responded to threats to validity. AK, MK, and RH performed the laboratory investigation. KJ, RT, and DM developed the analytical approach, conducted the statistical analysis, constructed the tables and figures, and helped interpret the results. All authors contributed to the article and approved the submitted version.
