# Supplementary material for: Faecal markers of intestinal inflammation in slum infants following yogurt intervention: A pilot randomized controlled trial in Bangladesh
Source: Front Microbiomes. 2023 Mar 23;2:1029839. doi: 10.3389/frmbi.2023.1029839 (PMC12993563; doi:10.3389/frmbi.2023.1029839)
Supplement: Supplementary file 1 [file DataSheet_1.docx]

Supplementary table 1: Consumption of food form different food groups by study arm (24 hours recall)

| Food groups | Baseline | 3-month follow up | N=127 | |
| --- | --- | --- | --- | --- |
|  | % | % | ^§^OR | 95% CI |
| **Breastmilk** | |  |  |  |
| Control | 100 | 97.7 | 1.00 | - |
| Education | 92.9 | 95.2 | - | - |
| Yogurt plus education | 92.9 | 95.2 | - | - |
| **Grains** | |  |  |  |
| Control | 37.2 | 97.7 | 1.00 | - |
| Education | 40.5 | 100.0 | - | - |
| Yogurt plus education | 45.2 | 95.2 | 0.32 | 0.02, 4.60 |
| **Flesh foods** | |  |  |  |
| Control | 11.6 | 41.9 | 1.00 | - |
| Education | 7.1 | 35.7 | 1.32 | 0.23, 7.49 |
| Yogurt plus education | 2.4 | 47.6 | 6.81 | 0.65, 71.70 |
| **Lentils** | |  |  |  |
| Control | 11.6 | 23.3 | 1.00 | - |
| Education | 16.7 | 45.2 | 2.01 | 0.39, 10.50 |
| Yogurt plus education | 9.5 | 42.9 | 3.59 | 0.60, 21.34 |
| **Eggs** | |  |  |  |
| Control | 4.7 | 20.9 | 1.00 | - |
| Education | 11.9 | 35.7 | 0.80 | 0.10, 6.70 |
| Yogurt plus education | 4.8 | 23.8 | 1.16 | 0.10, 13.74 |
| **Milk products** | |  |  |  |
| Control | 58.1 | 46.5 | 1.00 | - |
| Education | 61.9 | 47.6 | 0.88 | 0.23, 3.32 |
| Yogurt plus education | 54.8 | 47.6 | 1.25 | 0.33, 4.68 |
| **Yellow fruits and vegetables** | |  |  |  |
| Control | 14.0 | 23.3 | 1.00 | - |
| Education | 11.9 | 42.9 | 3.52 | 0.64, 19.48 |
| Yogurt plus education | 7.1 | 52.4 | **10.19** | **1.50, 69.04** |
| **Others fruits and vegetables** | |  |  |  |
| Control | 23.3 | 65.1 | 1.00 | - |
| Education | 31.0 | 78.6 | 1.38 | 0.31, 6.19 |
| Yogurt plus education | 26.2 | 83.3 | 2.57 | 0.53, 12.33 |

^§^Odds Ratio estimated using Logistic Mixed Model. Missing estimates are due to convergence error.

Supplementary table 2: Association between fecal biomarkers concentration and LAZ at 3-month follow up (full sample)

| Categories of log concentration of biomarkers | LAZ at 3-month follow up  N=127 | | |
| --- | --- | --- | --- |
|  | ^‡^RR | 95% CI | *p*- value |
| *Alpha 1 antitrypsin (AAT)* | | | |
| <25 percentile | 1.00 | - | - |
| 25-75 percentile | 0.79 | 0.60, 1.04 | 0.10 |
| >75 percentile | 0.83 | 0.61, 1.14 | 0.24 |
| *Myeloperoxidase (MPO)* | | | |
| <25 percentile | 1.00 | - | - |
| 25-75 percentile | 0.99 | 0.76, 1.29 | 0.96 |
| >75 percentile | 0.84 | 0.61, 1.16 | 0.28 |
| *Neopterin (NEO)* | | | |
| <25 percentile | 1.00 | - | - |
| 25-75 percentile | 0.79 | 0.61, 1.03 | 0.08 |
| >75 percentile | 0.75 | 0.55, 1.01 | 0.06 |
| *Composite score of EED* | | | |
| Lowest tertile | 1.00 | - | - |
| Middle tertile | 0.83 | 0.63, 1.08 | 0.17 |
| Highest tertile | 0.82 | 0.63, 1.07 | 0.14 |

^‡^Response Ratio (RR) was calculated using ANCOVA adjusted for baseline value of LAZ, child gender, household wealth, fever, diarrhea, acute respiratory infections, antibiotic consumption, number of illness tracking visits, exclusive breastfeeding status, mode of delivery, mother’s education, and minimum dietary diversity score

Supplementary table 2a: Association between fecal biomarkers concentration and LAZ at 3-month follow up by study group

|  | LAZ at 3-month follow | | | | | | | | |
| --- | --- | --- | --- | --- | --- | --- | --- | --- | --- |
| *Categories of log concentration of biomarkers* |  | Control N=43 |  |  | Education N=42 |  | Yogurt + education  N=42 | | |
|  | ^‡^RR | 95% CI | *p*-value | ^‡^RR | 95% CI | *p*-value | ^‡^ RR | 95% CI | *p*-value |
| *Alpha 1 antitrypsin (AAT)* | | |  |  |  |  |  |  |  |
| <25 percentile | 1.00 | - | - | 1.00 | - | - | 1.00 | - | - |
| 25-75 percentile | 0.79 | 0.41, 1.52 | 0.46 | 1.08 | 0.68, 1.71 | 0.74 | **0.59** | **0.37, 0.94** | **0.03** |
| >75 percentile | 1.10 | 0.49, 2.48 | 0.81 | 1.00 | 0.60, 1.67 | 1.00 | **0.47** | **0.28, 0.81** | **0.01** |
| *Myeloperoxidase (MPO)* | | |  |  |  |  |  |  |  |
| <25 percentile | 1.00 | - | - | 1.00 | - |  | 1.00 | - | - |
| 25-75 percentile | 1.21 | 0.55, 2.67 | 0.62 | 1.20 | 0.72, 2.01 | 0.47 | 1.16 | 0.68, 1.98 | 0.56 |
| >75 percentile | 0.80 | 0.30, 2.17 | 0.65 | 1.04 | 0.64, 1.69 | 0.87 | 0.94 | 0.52, 1.72 | 0.84 |
| *Neopterin (NEO)* | | |  |  |  |  |  |  |  |
| <25 percentile | 1.00 | - | - | 1.00 | - |  | 1.00 | - | - |
| 25-75 percentile | 0.55 | 0.23, 1.31 | 0.17 | 0.94 | 0.60, 1.50 | 0.80 | 0.79 | 0.47, 1.35 | 0.38 |
| >75 percentile | 0.51 | 0.20, 1.30 | 0.15 | 1.71 | 1.07, 2.73 | 0.03 | 0.68 | 0.40, 1.17 | 0.16 |
| *Composite score of EED* | | |  |  |  |  |  |  |  |
| Lowest tertile | 1.00 | - | - | 1.00 | - |  | 1.00 | - | - |
| Middle tertile | 0.60 | 0.33, 1.08 | 0.09 | 1.08 | 0.68, 1.71 | 0.74 | 0.85 | 0.51, 1.43 | 0.53 |
| Highest tertile | 0.54 | 0.26, 1.10 | 0.09 | 1.26 | 0.84, 1.90 | 0.25 | 0.83 | 0.48, 1.43 | 0.49 |

^‡^Response Ratio (RR) was calculated using ANCOVA adjusted for baseline value of LAZ, child gender, household wealth, fever, diarrhea, acute respiratory infections, antibiotic consumption, number of illness tracking visits, exclusive breastfeeding status, mode of delivery, mother’s education, and minimum dietary diversity score

Supplementary table 3: Association between fecal biomarkers concentration at 3-month follow up and yogurt consumption

| Compliance with the yogurt intervention  N=42 | Log AAT concentration | Log MPO concentration | Log NEO concentration |
| --- | --- | --- | --- |
|  | ^‡^RR (95% CI) | ^‡^RR (95% CI) | ^‡^RR (95% CI) |
| <80% (n=8) | 1.00 | 1.00 | 1.00 |
| ≥80% (n=34) | 0.63 (0.38, 1.03)  *p*-value 0.07 | 0.89 (0.30, 2.62)  *p*-value 0.83 | 0.80 (0.48, 1.34)  *p*-value 0.39 |

^§^≥80% compliance with intervention

^‡^Response Ratio (RR) was calculated using ANCOVA adjusted for baseline value and minimum dietary diversity score

Supplementary Figure 1: Six basic steps of making yogurt at home
